# The lived experiences of oropharyngeal dysphagia in adults living with fibromyalgia

**DOI:** 10.1111/hex.13932

**Published:** 2023-12-07

**Authors:** Órla Gilheaney, Joeann Hussey, Kathleen McTiernan

**Affiliations:** ^1^ Department of Clinical Speech and Language Studies Trinity College Dublin Dublin Ireland; ^2^ School of Linguistic, Speech, and Communication Sciences Trinity College Dublin Dublin Ireland; ^3^ Present address: Assistant Professor, Trinity College Dublin Dublin Ireland

**Keywords:** dysphagia, fibromyalgia, lived experience, psychosocial, qualitative research

## Abstract

**Background:**

Fibromyalgia is a chronic pain condition which has recently been linked with eating, drinking and swallowing difficulties (dysphagia). However, to date, sample sizes within completed research are small and study designs heterogeneous, and therefore, little is known about the lived experiences of dysphagia among people with fibromyalgia. To go some way towards addressing this gap in the literature, this study collected and analysed the first‐hand experiences of the physical symptoms, the psychosocial impacts and environmental factors that influenced the lived experience of a sample of people living with fibromyalgia‐associated dysphagia.

**Methods:**

Qualitative semi‐structured interviews were conducted with adults with dysphagia and fibromyalgia. Reflexive thematic analysis was employed and themes were identified regarding the reported experience and impact of swallowing problems. The same researcher conducted the interviews and extracted all data, and a second researcher analysed a random sample of 5% of the data for accuracy, with no disagreements arising between the two researchers.

**Results:**

All participants (*n* = 8) reported the negative psychosocial impact of their dysphagia. Participants reported managing their dysphagia independently, primarily using compensatory strategies. Participants discussed feeling unsupported in healthcare interactions due to clinicians not understanding the occurrence, nature or impact of eating, drinking and swallowing difficulties. Participants also reported that they did not have access to evidence‐based management strategies that adequately addressed their fibromyalgia‐related swallowing problems.

**Conclusions:**

Despite minimal previous research in this area, findings here highlight the impact that dysphagia has on people with fibromyalgia. A broad range of physical symptoms were reported to have negative consequences across both social and emotional domains. The reported symptoms often required complex coping strategies and sometimes impeded participants from seeking suitable medical intervention from healthcare providers. There are both broad‐ranging implications of fibromyalgia‐associated dysphagia and reported poor perceptions of medical interactions for this cohort of patients. Therefore, there is evidently a need for clinical research into the management of this condition to develop patient‐centred care delivery options and to equip healthcare professionals with the knowledge and skills necessary to provide efficacious management to this group.

**Patient or Public Contribution:**

Before initiation of the qualitative interviews, the interview schedule was piloted with an individual living with fibromyalgia and dysphagia, with feedback provided on the appropriate wording and format of semi‐structured questioning.

## 1 INTRODUCTION

### 1.1 Fibromyalgia

Fibromyalgia is a condition characterised by chronic pain, tenderness of the musculoskeletal system, fatigue and sleep disturbances,^1^ which affects up to 8% of the general global population.^2^, ^3^, ^4^, ^5^, ^6^ People with fibromyalgia may also experience cognitive and psychiatric symptoms, in addition to sensitivity to external stimuli.^7^ This clinical group is often diagnosed with multiple additional comorbidities, commonly including temporomandibular disorders (TMDs), migraine and irritable bowel syndrome (IBS).^8^

### 1.2 Dysphagia

Dysphagia is a condition characterised by difficulty swallowing across the oral, pharyngeal, or oesophageal phases, which may be caused by a range of idiopathic or acquired conditions.^9^, ^10^ It can occur across the lifespan and can significantly impact a person's health, leading to adverse and potentially fatal consequences if left untreated, including malnutrition, dehydration and pneumonia.^11^, ^12^ In addition to these physical consequences, dysphagia can also have a negative impact on quality of life.^9^ In other neurological populations, such as those living with motor neuron disease or Parkinson's disease, research has shown that dysphagia often prompts negative food perceptions. This can subsequently reduce an individual's pleasure of eating due to the consequences of the swallowing problems.^13^, ^14^

### 1.3 Fibromyalgia associated‐dysphagia—Presentation and prevalence

Dysphagia is not currently classified as a feature of fibromyalgia by the reference standard American College of Rheumatology criteria^15^ or other existing diagnostic systems, such as the ACTTION‐APS Pain Taxonomy.^16^ However, despite this lack of recognition, there is emerging research regarding the experience of dysphagia among those living with fibromyalgia. For example, Seccia et al.^17^ discussed that patients with fibromyalgia may present with symptoms of oral–oesophageal pain and increased sensitivity in this area. Furthermore, Rhodus et al.^18^ outlined that dysphagia can occur in conjunction with other oral symptoms in this population. These symptoms include xerostomia, glossodynia dysgeusia and odynophagia, a point which was also emphasised by De Baat et al.^19^ These studies highlight the currently ambiguous nature of the relationship between fibromyalgia and dysphagia, and the need for further investigation into the causation and presentation of this condition. The sparse amount of research into this topic was re‐emphasised recently in a systematic review, which found a very limited number of available studies which investigated dysphagia in those with fibromyalgia.^20^ Within the identified studies, meta‐analysis found dysphagia in 51.9% of participants, while gastro‐oesophageal reflux was reported in 25.9%. However, the prevalence of other reported symptoms (e.g., glossodynia, dysgeusia, odynophagia, xerostomia or sensation of choking/bolus sticking in the pharyngeal region) was reported in too few studies to allow for synthesis in data analysis. Furthermore, the prevalence of aspiration/penetration on food/liquids/secretions or impaired mastication or pain or fatigue on chewing were not reported in any studies, thus hindering true understanding of these issues.^20^ Despite the small amount of information on the co‐occurrence of these conditions, there are ample anecdotal reports within online message forums (e.g., Reddit) of patients living with distressing oropharyngeal dysphagia symptoms, which they describe as disabling and disruptive.^21^

### 1.4 Fibromyalgia associated‐dysphagia—Hypothesised aetiology

There is no current definitive evidence regarding the causation of dysphagia in those living with fibromyalgia. There are, however, hypotheses that some of the commonly prescribed pain medications for this condition may secondarily cause oropharyngeal issues.^22^ Other potential aetiologies have been suggested regarding altered sensitivities in the central and peripheral nervous system or altered physiology in the muscles associated with swallowing secondary to neurological changes in the central nervous system.^20^ However, there is no definitive evidence proving aetiology at present and research in this field is sparse.

### 1.5 Fibromyalgia associated‐dysphagia—Research gap

People with fibromyalgia who are diagnosed with additional conditions (e.g., TMDs, IBS, rheumatoid arthritis) are reported to experience poorer health‐related quality of life with worse psychosocial and overall health outcomes than those who live with fibromyalgia alone.^23^ Although there are emerging scientific reports regarding the prevalence^20^ and impact^24^ of dysphagia in those living with fibromyalgia, there is little in‐depth knowledge available regarding the full spectrum of oropharyngeal symptoms and their psychosocial impact. As a result, the provision of effective clinical management for this cohort of patients is lacking. Therefore, in an attempt to initiate research in this area, which may contribute to future positive developments in clinical practice, this study investigated the physical psychosocial implications of fibromyalgia‐associated dysphagia. Furthermore, the strategies used by the participants to cope with the reported challenges on a daily basis were also investigated.

## 2 MATERIALS AND METHODS

This qualitative research study adopted a phenomenological perspective. Data collection was carried out through semi‐structured online interviews with people with fibromyalgia and oropharyngeal dysphagia, with ethical approval granted by Trinity College Dublin (Applicant code TT59). Semi‐structured individual interviews allowed for in‐depth and flexible investigation of personal experience,^25^, ^26^ which is appropriate in cases such as this, when minimal information is known about a condition and the experiences of those living with the issues themselves is sought in their capacity as the experts in the condition.

An interview guide (Table 1) was created by the second author based on studies with a similar design^27^ and piloted with an individual with fibromyalgia and dysphagia, with feedback provided regarding the clarity of wording within the original semi‐structured questions, resulting in minor rephrasing.

**Table 1:** Interview guide.

Interview topic area	Prompt/sample questions
Interview opening	Outline the layout of the interview, the projected time interview will take, ask the participant if they have any questions and obtain verbal consent.
Demographic questions	Age, gender, country of origin, confirmation of clinical diagnosis of fibromyalgia, clinical diagnosis of dysphagia or self‐diagnosis of dysphagia.
Physical symptoms	Can you describe any difficulties that you experience with your eating, drinking and swallowing?
	Follow up questions related to response of initial question.
Emotional impact of feeding, eating, drinking and swallowing	How do the problems with swallowing that you are describing make you feel?
	Follow up questions related to response of initial question.
Social impact of feeding, eating, drinking and swallowing	Do your swallowing difficulties affect how you socialise with others?
	Do your swallowing difficulties affect who you socialise with (if so, how)?
	Follow up questions related to response of initial questions.
Support network	Is there anyone in your life who supports you with your swallowing problems (other people who also have Fibromyalgia, healthcare staff, family, friends)?
	Do you feel like people in your life understand your swallowing problem?
	How do you cope with your dysphagia?
	What do you do that helps you with your swallowing problem?
	Follow up questions related to response of initial questions.
Interview closing	Summary of what was covered during the interview.
	Ask if participant has any questions.
	Explain the next steps (e.g., participant will have opportunity to check transcription of interview),
	Thank participant for participation in the study,

Participants who met the eligibility criteria (Table 2) were recruited from charities and organisations based in Ireland and the United Kingdom dedicated to fibromyalgia and other rheumatic conditions.

**Table 2:** Inclusion and exclusion criteria.

Inclusion criteria	Exclusion criteria
People with a diagnosis of fibromyalgia from a licensed medical practitioner. People with self‐identified or clinically diagnosed eating, drinking and swallowing problems. People with sufficient English language skills to partake in an interview.	People under the age of 18. Adults with a learning disability. Adults with language or cognitive‐communication difficulties that would preclude them from partaking in the interview.

Administrators in these organisations acted as gatekeepers in disseminating both the participants' information leaflet and a prepared letter to all the members of their organisation. Individuals who wished to participate contacted the principal researcher and were given a consent form to sign, forming a voluntary response sample.

Interviews, lasting between 30 and 60 min in duration, were conducted over Zoom (zoom.us) by the second author, who was a final year speech and language pathology student completing their dissertation at the time. Each interview was audio‐visually recorded. The second author then conducted broad orthographic transcription, with the subsequent member of the research team checking the transcripts to ensure accuracy. The transcripts were analysed by the second author using reflexive thematic analysis, following the six steps outlined by Braun and Clarke^28^, ^29^ to identify themes and subthemes in the data. To account for the reliability and validity of the data analysis, the first author analysed a random selection of 5% of the data collected, comparing the selection with original transcriptions for verification of accuracy. There was full consensus on transcription content between the two researchers.

## 3 RESULTS

### 3.1 Participant demographics

Seventeen volunteers initially expressed interest in the study, and as nine people withdrew from the study before data collection, the final sample size was eight participants.

All of the participants identified as female (*n* = 8) and were from either the United Kingdom (87.5%, *n* = 7) or the Republic of Ireland (12.5%, *n* = 1). The mean age of the participants was 54.83 with a range of 47–59 (Table 3). Half of the participants received a diagnosis of fibromyalgia between 2 and 8 years before the time of data collection (2 years *n* = 2, 5 years *n* = 1, 8 years *n* = 1) and four participants had received a diagnosis of dysphagia more than 10 years previously. The length of time since onset of dysphagia for the cohort ranged from 2 to 5 years for most of the participants (2 years *n* = 3, 3 years *n* = 1, 5 years *n* = 1), however for three participants the length of time since the onset of dysphagia was greater than 5 years.

**Table 3:** Description of sample.

Mean age and age range	54.83 years (age range = 47–59)
Gender	100% Female
Countries of origin	United Kingdom = 88%, Republic of Ireland = 12%
Clinical diagnosis of fibromyalgia	100%
Diagnosis of dysphagia	Clinical diagnosis = 12%, self diagnosis = 88%

### 3.2 Thematic analysis

Reflexive thematic analysis yielded five primary themes from the data, and a further 23 subthemes.

#### 3.2.1 Theme 1: Physical symptoms of dysphagia

Participants reported oral, pharyngeal and oesophageal phase dysphagia symptoms (Table 4).

**Table 4:** Physical symptoms of dysphagia.

Symptom	Participant quote
Oral symptoms of dysphagia (38% of participants reported some symptoms under this theme)	
Reduced tongue movement	‘I can't physically make my tongue work’. (P2)
Difficulty initiating a swallow	‘Sometimes I can't find a swallow, which sounds really weird’. (P4)
Xerostomia	‘The tongue and the whole area gets very dry’. (P6)
Pharyngeal symptoms of dysphagia (100% of participants reported some symptoms under this theme)	
Odynophagia	‘It can be, sometimes, quite a painful thing … It's like something was stuck but would hurt like’. (P1)
Difficulty swallowing food	‘While you're eating or something, you know, it's always going down the wrong hole type thing’. (P2)
Difficulty swallowing fluids	‘Every time I drink something, I'll cough’. (P8)
Difficulty swallowing medication	‘If the doctor gives me a medication that's big, like a big paracetamol, I won't swallow that down’. (P6)
Difficulty swallowing saliva/managing secretions	‘I can choke on anything, just, it's very difficult to explain it. It can be a tiny droplet of water, it can be saliva, it can be I'm, like, really choking’. (P3)
Choking episodes	‘My husband had to do the Heimlich on me about three, four times. He had to do the Heimlich on me because the choking has gotten so bad that I just, I couldn't breathe’. (P8)
Oesophageal symptoms of dysphagia (100% of participants reported some symptoms under this theme)	
Reflux	‘I do have reflux. I have medication for that’. (P5)
Oesophageal dysmotility	‘I had some swallowing tests done. And they found that I've got something called dysmotility of the oesophagus’. (P4)

Several symptoms of oral phase dysphagia were reported, including poor tongue control and xerostomia. Three participants experienced difficulties initiating a swallow and they found it challenging to explain this concept as they thought that this was unusual:

So, I'm here having a cup of coffee and my husband comes in to ask me a question. I've got a mouthful of coffee and I couldn't swallow it for love nor money.

Regarding the pharyngeal phase of the swallow, all participants reported experiencing difficulties swallowing food of varying textures and consistencies and thin fluids. More than half of the interviewees described swallowing tablets:

And the oval paracetamol capsules are about the biggest I can take, anything bigger than that I would have to break up because it's it I just can't do it. I would be coughing it back out. (P6)

Participants also explained that they struggle to manage swallowing their secretions:

Or even saliva sometimes, I can't even swallow saliva. It's as if I can't control the back of my throat and my tongue. (P2)

The interviewees also detailed reports of choking episodes, with odynophagia also described by three participants.

Finally, several participants reported oesophageal symptoms. Five participants described experiencing acid reflux and several people also reported symptoms of oesophageal dysmotility.

#### 3.2.2 Theme 2: Social issues associated with reported experience of dysphagia

The physical symptoms described above were associated with subsequent social impacts for all of the participants in the study (Table 5).

**Table 5:** Social issues associated with reported experience of dysphagia.

Social issue	Participant quote
Avoiding social interactions	‘I can't drink any alcohol. I can't enjoy life, particularly I can't go out for dinners’. (P4)
Limited choice on a restaurant menu	‘I couldn't go for a burger in a sit‐down situation, because, that, I'm guaranteed now, if I bought this big burger, I'd be guaranteed I'd have trouble getting it down’. (P1)
Being conscious of their swallowing issues when eating in a restaurant	‘When I go somewhere, I always explained to people, look, don't worry, I will cough and I might choke a bit, but it's part of my condition and I can't help it. I try not to, but you know, I'm sorry if I do and it upsets you or embarrasses you or whatever’. (P5)

Each participant discussed that they avoided social interactions, both public and familial:

It makes me feel like I don't want to go out for a meal. Going over to my son's house. I've got a grandson who lives with my son. And I'm not wanting to frighten them in case I start to choke. (P3)

Many participants reported that their ability to order from a set menu is impacted. Some participants describe the foods they order as:

Softer food, pasta, and things like that, where I don't have to, it's going to sound awful, where it slides down easily. (P6)

Others stated that they do not eat restaurant food at all (e.g., ‘Whenever I go anywhere, I take my own food’ [P7]).

Three participants reported that they need to plan for the possibility that they will experience swallowing problems while socialising. These considerations involved the specific location of their seating:

And I'll try and sit in a corner where I can hiccup as quietly as possible so that, you know, that I'm not surrounded by everyone listening. (P1)

And their proximity to a private bathroom:

But in my own head I would, you know, have everything planned, just in case that episode started I could get there quickly. (P6)

#### 3.2.3 Theme 3: Emotional impact of the physical and social issues associated with experience of dysphagia

All of the participants reported that they had experienced emotional challenges associated with having fibromyalgia‐associated dysphagia. The emotional impacts are divided into two sections that focus on the physical (Table 6) and social aspects (Table 7) of dysphagia.

**Table 6:** Emotional impact of the physical issues associated with reported experience of dysphagia.

Emotion described	Participant quote
Anxiety/fear of choking	‘But it does worry me because if I choke when I'm on my own, and I do stop breathing, or I inhale the drink rather than swallow the drink’. (P5)
Lack of enjoyment from food	‘I'm eating to live, I don't, enjoy, I don't, it's not an enjoyable experience to eat. I know I have to because I know I won't survive without it’. (P7)
Self‐doubt	‘I was blaming myself, I would be like, Jesus, if I wasn't such a pig, now and ate a bit slower this mightn't happen’. (P1)

**Table 7:** Emotional impact of the social issues associated with reported experience of dysphagia.

Emotion described	Participant quote
Embarrassment	‘And I was so embarrassed because everybody was looking at me’. (P8)
Fear of being perceived to have swallowing problems	‘We don't really eat out a lot. Mainly because I'm worried about choking in, in, in public. And, you know, I have done, a couple of times, and it's, I've had to go to the bathroom. And the waitresses have followed me’. (P6)

Anxiety was the prevailing emotion related to the physical aspects of having dysphagia described by the participants. All participants described feeling anxious about eating, with the severity of this ranging from mild nervousness to severe fear of swallowing, particularly when eating alone:

I'm terrified of eating. My husband sometimes goes away for the weekend to see his friends and when he's away, I barely eat anything. (P8)

One participant reported seeking psychological intervention to ameliorate this anxiety (e.g., ‘I had to go to cognitive behavioural therapy, to just to try and help me to swallow fluids’ [P7]). These feelings of anxiety rendered a lack of enjoyment from eating for more than half of the participants, to the point that they will skip meals and eat less than advised:

I sometimes just won't even bother with an evening meal, if I'm doing something for everybody else, that is, like potatoes I struggle with, like if I make wedges, or something, I don't really fancy them much anymore. So, if I'm making something for everybody else, I sometimes just won't even bother or pick bits out, you know, and I don't have the whole meal. (P2)

A small cohort of participants (*n* = 3) felt a degree of doubt that they were to blame for their swallowing problems. After they described their physical symptoms, some participants would follow up with hypotheses that they were causing their problems (e.g., ‘So I don't know whether that's anything to do with that or if I'm drinking too fast or anything’ [P2]). These participants felt in some way responsible for their physical symptoms which would lead to further negative emotions (Table 7).

All of the participants reported that their swallowing problem had an emotional impact on them, however, the emotional impact of their experiences of the swallowing problems differed if they were discussing eating or drinking in a social situation. They described feeling embarrassment and shame if they experienced problems swallowing food or drink in a restaurant or at a social gathering. The embarrassment that the participants reported translated into fear for some. However, rather than a fear of choking, they had a fear of being perceived to have swallowing difficulties by unfamiliar people:

We went away a few weeks ago and went to a fish restaurant. I ended up not finishing my meal, really because I was so scared of, you know, that I'm in a restaurant, what if I end up choking. (P2)

Participants felt anxiety about having difficulties swallowing in public, as this would lead to further embarrassment:

I'm very conscious, because it's embarrassing, you know, I don't want to have a coughing fit, where I end up literally in tears from the choking, and you know, all your makeup washes down your face and you look like an imbecile, you know. So it does, it just limits life. (P5)

#### 3.2.4 Theme 4: Experiences in healthcare

Participants also discussed their attempts to access help and medical support for their swallowing problems (Table 8).

**Table 8:** Experiences in healthcare.

Experience	Participant quote
Feeling dismissed and unheard	‘When I first started it. I told him, I was like, I can't swallow. And he's like, just relax. You know, and he's like, don't make it a habit, is what I got told’. (P8)
Lack of awareness of dysphagia in fibromyalgia	‘Now, I did ask the question of my GP some time ago. And he kind of, he didn't think there was any connection, he kind of shrugged it off, but I suppose he just didn't hear it before’. (P1)
Lack of follow‐up with patients	‘No, no, nothing else. They've basically, they've just said, it's, you know, it's just one of those things getting older. Everything's, oh, you're getting a bit old, and then you've got expect some weird things to happen’. (P6)
Limited SLT assessment and intervention	‘She sent me a letter, and it's got some exercises on it for me to do with my tongue’. (P3)
Impact on patients' self‐esteem	‘Oh god yeah, so it just knocks your confidence a bit’. (P2)
Avoidance of further healthcare interactions	I've done all this myself because I haven't really had that much support from anybody, I have to say apart from me’. (P4)

Abbreviation: GP, general practitioner.

Three quarters reported that they sometimes did not feel listened to in healthcare interactions (*n* = 6). They described feeling dismissed when seeking help from medical professionals. In healthcare interactions, participants also felt that their healthcare providers were not aware of the occurrence of dysphagia in people with fibromyalgia. After these healthcare exchanges, there was sometimes a lack of follow‐up care, and no further investigation into their dysphagia was offered:

The doctor when he spoke to me, he said I think you've got a funny thing going on with your throat mechanism. He said I think it opens and closes the wrong way. Now, that's never been tested. (P5)

Three participants reported receiving speech and language therapy input for their swallowing difficulties. They all had clinical assessments, but no instrumental evaluations were carried out. One participant was given no further follow‐up (e.g., ‘Nothing really happened with that. That meeting at all, nothing really came of it’ [P8]), and two were given an exercise regime to complete.

These subthemes had a negative impact on the participant's self‐esteem. The participants expressed that they felt more concern and uncertainty after reaching out for support. These feelings of uncertainty deterred the participants from engaging with healthcare providers for their swallowing difficulties, and for other issues relating to their fibromyalgia.

#### 3.2.5 Theme 5: Coping strategies

The final theme identified in the data was coping strategies. All of the participants reported that they used various coping strategies and that their support networks also used coping strategies to mitigate the challenges posed by having a swallowing problem. The coping strategies, therefore, are reported in the following two categories: strategies used by the participants themselves and strategies that are facilitated by their support networks (Tables 9 and 10).

**Table 9:** Self‐facilitated coping strategies.

Strategy	Quote from participant
Reducing size of bolus and meals	‘I now don't eat big meals, I do more, more grazing’. (P2)
Restricting and modifying diet	‘I've stopped eating certain foods. So, like apples. I don't eat those. Because, that, I've gotten those stuck before in the past’. (P8)
Using consistent liquid washes	‘I've always got to have a drink at hand’. (P3)
Using calming strategies	‘I've learned now, although it's extremely difficult to do, I really have to stop. And I just stop and stand still. And I try to breathe’. (P8)

**Table 10:** Coping strategies facilitated by others.

Strategy	Participant quote
Helps the participant to use individual strategies	‘If I need a drink. I've always I've always got a bottle of water beside me when I'm having something to eat. But my daughter, she's 23. She's always very aware’. (P3)
Being present at mealtimes	‘With family, with, you know, they're aware that I can have a coughing fit. So, they just, they just, they're there. They don't necessarily help me because I just have to wait for it to stop. But they're there in case anything happens, you know, and I don't happen to catch my breath, you now’. (P6)

Participants frequently avoided seeking further medical input in favour of managing their difficulties independently. This involved trial and error with compensatory strategies, for example, adapting what and how they ate and drank. Participants often controlled the bolus size to ensure it was small enough for them to swallow safely and they reduced the size of their meals.

I haven't had a proper full meal like that, call it a dinner plate size meal for about a year and a half. (P4)

Participants described restricting their diets to foods that they feel more comfortable eating. Carrying out consistent liquid washes of water relieved symptoms of xerostomia and helped the participants to swallow safely. Participants discussed that they use calming strategies when they are experiencing swallowing difficulties.

Participants' families and friends were reported to support them emotionally, eg:

My mum will just tell me to relax, or you know, if I was with her, she'd make me a drink, probably a hot drink and sit me down. (P5)

Participants also felt supported by their families if they were simply present while they were eating, as this mitigated any fear they felt about choking.

## 4 DISCUSSION

Each participant reported their own unique combination of physical symptoms of dysphagia, with varying degrees of severity, frequency and duration. All participants described the negative psychosocial impact of their dysphagia, in addition to reports of frequent unsatisfactory clinical encounters when seeking medical care.

### 4.1 Physical, social and emotional implications of fibromyalgia‐associated dysphagia

The psychosocial impact of swallowing problems was unmistakable in this study, resembling reports of dysphagia in other clinical populations.^9^, ^30^ As separate conditions, fibromyalgia and dysphagia can cause adverse mental health consequences.^31^, ^32^ When researchers analysed the negative psychological experiences reported, it was apparent that feelings of anxiety and panic may exacerbate the swallowing difficulties reported. It is possible that there is a cycle occurring in the lives of the participants whereby their physical swallowing problems cause feelings of fear and anxiety, and that these emotions cause their physical symptoms to worsen or become more prolonged. This connection identified here between anxiety‐intensifying symptoms has already been reported in the literature. A population‐based study carried out by Csupak et al.^33^ concluded that people experiencing chronic pain have a higher prevalence of generalised anxiety disorder, and that people with anxiety report more severe pain and higher levels of disability Participants' emotions related to eating and drinking were often negative here. Reports of feeling shame and embarrassment when eating in a public space were unequivocal, and there was a palpable fear of being perceived as having a swallowing problem when participants discussed eating around unfamiliar people, similar to previous studies on dysphagia in other clinical cohorts.^34^, ^35^, ^36^, ^37^ The psychological impact of dysphagia in those living with fibromyalgia‐associated dysphagia was heightened in social situations, as reported in this study. Extrinsic factors can play a role in a fear of socialising. Public awareness of dysphagia is circumscribed, and personal bias could lead to an individual being treated differently if they are perceived to have problems eating or drinking.^34^, ^38^ A survey conducted to establish public awareness of dysphagia found that 71% of the sample did not know about the condition.^39^ Fibromyalgia and dysphagia are invisible illnesses, which can lead to people feeling isolated and stigmatised. It is evident that public perceptions of these conditions contribute to the negative psychosocial impact on this group of people. These findings highlight a need for people with dysphagia and fibromyalgia to be offered support from professionals who understand the physical, emotional and social factors that are impacted by swallowing difficulties.

### 4.2 Experiences in healthcare

Similar to previous work in the field of fibromyalgia,^40^ interviewees here expressed that they sometimes did not feel listened to in healthcare interactions and that follow‐up support was rare. Some participants reported that the healthcare professionals were not aware of dysphagia occurring in people with fibromyalgia. This is understandable as there is very little existing academic information about these conditions co‐occurring. Therefore, clinicians may not be currently aware of their role in supporting patients in this cohort.

Recent studies into the multidisciplinary management of fibromyalgia highlight the importance of multiple disciplines supporting individuals with this condition. Rasmussen et al.^41^ found that a multidisciplinary team (MDT) helped to influence the self‐efficacy of participants, and they highlighted new strategies that could be implemented to improve patient quality of life. Researching a condition through a unidisciplinary lens, particularly with a condition that is so varied, means that although an in‐depth investigation of specific symptoms can be carried out, it may result in possible explanations of the findings to be missed. An article published on the diet of people with fibromyalgia and the associated psychosocial outcomes concluded that a poor diet and suboptimal food intake is linked to negative effects on psychological well‐being.^24^ Results here show that the participants often eliminated foods from their diet and, in some cases, avoided meals altogether to feel more secure when eating. These findings highlight the importance of multidisciplinary management of all symptoms associated with fibromyalgia, including swallowing difficulties. If people with fibromyalgia could be offered specialised support and evidence‐based intervention plans that target the range of physical, psychosocial and occupational issues that they experience, then the burden of having to manage their condition in isolation could be reduced and their sense of well‐being could be increased.

### 4.3 Independent management of symptoms

Perhaps as a consequence of the negative experiences with health professionals discussed above, many participants here reported self‐managing their dysphagia. Self‐management of dysphagia is not uncommon in other clinical populations,^14^ and all participants here independently reported adjusting their lives to cope with their swallowing problems. Many of the participants reported avoiding social interactions, while others used relaxation techniques when they experienced swallowing problems, and many resorted to even eliminating various food types from their diet. The findings here mirror results produced in previous research from other cohorts, suggesting a shared dysphagia experienced across populations.^42^ Strategies facilitated by participants' family and friends, such as simply being present when a participant was eating or drinking, were reported to be helpful. It was notable, however, that these coping mechanisms were often extensions of the strategies used by participants themselves which indicates that the people in this sample mostly managed their swallowing difficulties in isolation. Therefore, this isolation highlights the potential for consequences of unmanaged fibromyalgia‐associated dysphagia (or dysphagia which is managed solely by a patient without clinical guidance) to negatively impact on an individual's health and wellbeing.

### 4.4 Limitations

Despite broad canvassing, the sample size for this study was small and homogeneous and therefore, the findings of future studies may differ depending on the diversity of the samples. In addition, completing the interviews online meant that some potential participants may have been indirectly excluded from the study due to limited technological knowledge. As such, in‐person interviews with a broader, more diverse, sample would be recommended to increase the generalisability of findings in future studies.

## 5 CONCLUSION

This qualitative study provides an insightful perspective on how people with fibromyalgia are affected by oropharyngeal dysphagia. The findings of this study exemplify that people with fibromyalgia are adversely affected by this condition, and often manage their condition in isolation. These findings indicate the need for increased MDT involvement in the holistic management of dysphagia in this client group, where their physical, social and emotional well‐being can be supported. Further research is needed to identify how members of the MDT can provide care for this client group, to address the full range of their difficulties and thereby improve their quality of life.

## AUTHOR CONTRIBUTIONS

**Órla Gilheaney**: Conceptualisation; supervision; methodology; writing—review and editing; formal analysis. **Joeann Hussey**: Investigation; writing—original draft. **Kathleen McTiernan**: Conceptualisation; methodology; formal analysis; writing—review and editing; supervision.

## CONFLICT OF INTEREST STATEMENT

The authors declare no conflict of interest.

## ETHICS STATEMENT

Ethical approval for this study was granted by the Trinity College Dublin School of Linguistic, Speech and Communication Sciences Research Ethics Committee (TT59). Informed consent was obtained from all individual participants included in the study.

## Data Availability

The data that support the findings of this study are available on request from the corresponding author. The data are not publicly available due to privacy or ethical restrictions.
